# The risk of exotic venomous snakes to public health in Brazil

**DOI:** 10.1590/0037-8682-0585-2020

**Published:** 2021-03-22

**Authors:** Paulo Sérgio Bernarde, Fan Hui Wen, Wuelton Marcelo Monteiro

**Affiliations:** 1 Universidade Federal do Acre, Campus Floresta, Centro Multidisciplinar, Laboratório de Herpetologia, Cruzeiro do Sul, AC, Brasil.; 2 Instituto Butantan, Núcleo Estratégico de Venenos e Antivenenos, São Paulo, SP, Brasil.; 3 Universidade do Estado do Amazonas, Escola Superior de Ciências da Saúde, Manaus, AM, Brasil.; 4 Fundação de Medicina Tropical Dr. Heitor Vieira Dourado, Diretoria de Ensino e Pesquisa, Manaus, AM, Brasil.

**Dear Editor:**

The hobby of breeding exotic snakes has generated a health problem, with numerous cases of snakebites around the world, mainly in North America and Europe; this is because some breeders are acquiring venomous species^1^ ^,^ ^2^ ^,^ ^3^ ^,^ ^4^. Between 2005 and 2011, 258 cases of snakebites involving 61 species of exotic venomous snakes were recorded in the United States, which is an average of 37 cases per year^3^. In northeastern Germany and southeastern France, from 1996 to 2006, 155 cases were recorded (an average of 14 per year)^2^. The monocled cobra (*Naja kaouthia*) is among the most bred species and a major cause of envenomations in the United States, including cases that have been fatal^1^ ^,^ ^3^. 

**FIGURE 1:**
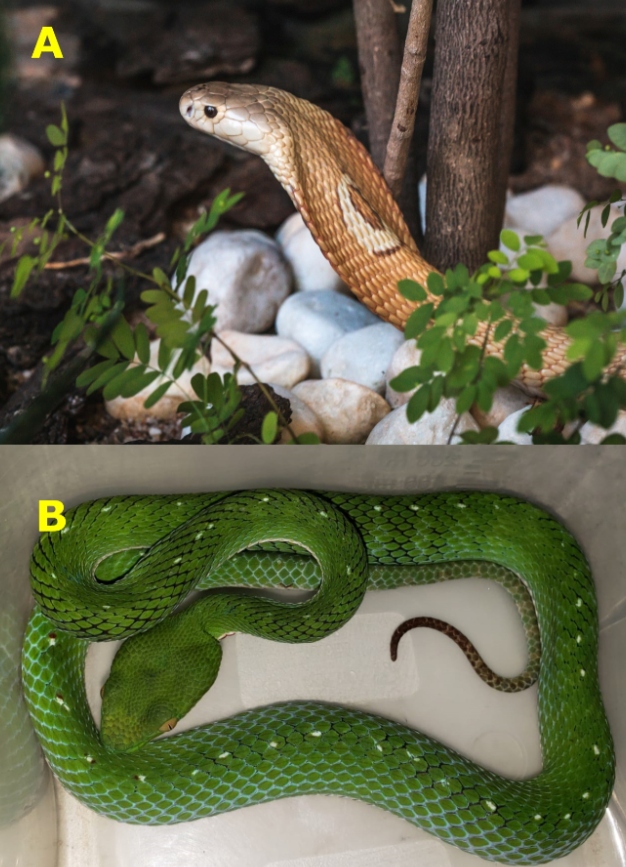
**(A)** Monocled Cobra (*Naja kaouthia*). Photo: Ivan Mattos; **(B)** Vogel's pit viper (*Trimeresurus vogeli*). Photo: Marco Freitas.

The monocled cobra (*N. kaouthia*) is a species that is native to Asia; it exists in several Asian countries including Bangladesh, Myanmar, Cambodia, India, Bhutan, Laos, Malaysia, Nepal, China, Thailand and Vietnam^5^, where snakebites involving the species are common^6^. It belongs to the family Elapidae, which also includes true corals from America, the taipan from Australia, mambas from Africa, and other species of cobras (“najas”) from Africa and Asia^5^. All species are well known for being highly dangerous. The English name of the monocled cobra refers to the pattern on its back, which is similar to an eye (monocle) (Figure 1A ); this species can reach approximately 1.5 m in length^5^. 

The venom of *N. kaouthia* consists of low-molecular-weight neurotoxins, which explains the relative rapidity in the clinical evolution of envenomations. Owing to its wide geographical distribution, the venom of *N. kaouthia* presents significant variability in its composition^7^, which may have repercussions in therapies using antivenom against the main toxins^8^. Envenoming is characterized by pain and edema, and extensive local necrosis may also arise. Systemic manifestations can appear quickly; they are characterized by neuromuscular blockade due to the venom’s action on peripheral nerve endings. Thus, the patient presents paralysis of different muscle groups, notably eyelid ptosis, ophthalmoplegia, and descending paralysis, which can reach the musculature of the rib cage. Deaths can thus occur due to respiratory failure, mainly as a result of delayed application of the antivenom or when the antivenom is unavailable^6^. 

In 2017, a specimen of *N. kaouthia* was captured in Camboriú, a city on the coast of Santa Catarina state, Brazil and sent to the Butantan Institute^9^. Recently, an illegal breeder in the Federal District, Brazil was bitten by his pet monocled cobra, while handling it^10^. In the absence of the specific antivenom for this species in the country's hospital units, serum ampoules produced by the Queen Saovabha Memorial Institute, Thailand were supplied by the Butantan Institute and airlifted to Brasilia for treatment of the patient. The patient presented edema and pain, as well as respiratory distress about 1 h 30 min after the bite. He was intubated and provided with mechanical ventilation. He remained in the ICU for 72 h, progressively improving until he was discharged 6 days after admission.

This incident led environmental agencies to seize other exotic snakes that were being bred illegally in the Federal District, which included one person handing over a Vogel’s pit viper (*Trimeresurus vogeli*)^10^ (Figure 1B ), a venomous species of the family Viperidae, also of Asian origin (Thailand, Cambodia, Laos, and Vietnam)^5^. In addition to these records, in social network groups, Magalhães and São Pedro^11^ found eight species of viperids (*Agkistrodon contortrix, Atropoides olmec, Bothriechis schlegelii, Crotalus triseriatus, Deinagkistrodon acutus, Trimeresurus trigonocephalus, T. vogeli* and *T. venustus*) on sale in Brazil. These data show a variety of venomous snake species, many from other continents, for which there is no antivenom in stock in the country. Moreover, this may just be the tip of the iceberg of a problem that may be increasing invisibly without the knowledge of environmental authorities and the health system.

In Brazil, legislation does not permit the import and breeding of exotic venomous snakes as a hobby (rearing as a pet)^10^ ^,^ ^11^. Nevertheless, this activity continues to occur, and the real dimension of this problem is not known. These records of exotic venomous snakes and the case of envenomation by a *N. kaouthia* have revealed not only an illegal trade in animals but also a risk to the health of the population. There are species of venomous snakes in our territory being bred illegally whose antivenom is usually unavailable in hospital units or possibly only available in limited doses in some institutions that keep some specimens for scientific or display purposes. Another aggravating factor is the fact that health professionals do not have experience in dealing with this peculiar situation. 

The antivenom produced in Brazil is effective for neutralizing the envenomations caused by snakes present in our fauna. Thus, even the elapidic antivenom intended for true corals, which belong to the same family as the najas (Elapidae), would have not been able to reverse the clinical situation because the toxin classes of these snakes are different. Even when Vital Brazil was a doctor, the principle of specificity in the treatment of snakebites was already known, making, to this day, the production of antivenoms a process that necessarily involves the existence of livestock of national fauna in the laboratories where antivenom is produced. 

However, it is not only antivenom therapy that can reverse the clinical picture of envenomations; there are complementary treatments that can contribute to the improvement of the patient. In the case of snakebites with postsynaptic neurotoxins, the use of anticholinesterases (neostigmine) may be an alternative, if the specific antivenomin is unavailable or if there is a delay in its administration, especially in cases where the patient requires mechanical ventilation^1^ ^,^ ^4^ ^,^ ^6^. 

In addition to private breeders in their homes and exotic venomous snakes, it is important to remember that there is also the possibility of native snakes of Brazil being bred in regions where they do not exist naturally, which can cause accidents. One such incident occurred at the Sorocaba Zoo with a South American bushmaster (*Lachesis muta*) that was kept at the zoo and bit a biologist while it was being fed^12^. Because this species does not naturally occur in the state of São Paulo, the patient was transported to the city of São Paulo, where he received antivenom treatment with bothropic-laquetic antivenom. 

Keeping an exotic venomous snake illegally in Brazil is a crime, and the unavailability of the specific antivenom puts people's lives at risk. How many other exotic venomous snakes from other continents (for which there is no antivenom in the country) are being bred clandestinely and, as such, offer a risk to public health? 

Therefore, the following is recommended:

- Conduct a survey of exotic snake species being kept in educational, research, and conservation institutions around the country;
- Ensure that institutions that maintain exotic snakes have sufficient quantities of antivenom in the case of snakebites;
- When training health professionals, include instructions on how to proceed in cases of envenomation caused by exotic venomous snakes;
- Demand that the relevant authorities urgently investigate the existence of this illegal market in Brazil in order to curb animal trafficking.
